# How Nano-ZnO Affect Tomato Fruits (*Solanum lycopersicum* L.)? Analysis of Selected Fruit Parameters

**DOI:** 10.3390/ijms25158522

**Published:** 2024-08-05

**Authors:** Katarzyna Włodarczyk, Beata Smolińska, Iwona Majak

**Affiliations:** 1 Institute of Natural Products and Cosmetics, Department of Biotechnology and Food Science, Lodz University of Technology, Stefanowskiego 2/22 Str., 90-537 Lodz, Poland; beata.smolinska@p.lodz.pl; 2 Institute of Food Technology and Analysis, Department of Biotechnology and Food Science, Lodz University of Technology, Stefanowskiego 2/22 Str., 90-537 Lodz, Poland; iwona.majak@p.lodz.pl

**Keywords:** nanoparticles, nano-ZnO, antioxidants, tomatoes, crop plants, allergens

## Abstract

Tomato (*Solanum lycopersicum* L.), as one of the most valuable horticulture crops, was chosen to investigate the effect of nanoparticles (NPs) in the form of nano-ZnO combined with conventional fertilizer on the quality of tomato fruits, including their antioxidant potential (total antioxidant activity, lycopene and β-carotene content), sugars content and allergenic potential (profilin and Bet v 1 content). Nano-ZnO was implemented during plant cultivation, applied by foliar spraying or directly via soil, at three different concentrations (50, 150 and 250 mg/L). The obtained results suggest that the usage of NPs during tomato plant cultivation had minor impacts on parameters such as total antioxidant activity or the content of selected allergens. Even though the total antioxidant activity was not affected by nano-ZnO, the malondialdehyde activity (MDA) content was notably decreased in fruits under nano-ZnO treatment. The content of lycopene and β-carotene was significantly affected by the use of nano-ZnO. Moreover, the usage of nano-ZnO significantly increased the total sugar content in fruits treated with nanoparticles via foliar spraying. Based on the obtained results, it can be stated that nano-ZnO, regardless of the method of application, significantly affected tomato fruits which can be beneficial for fruit production.

## 1. Introduction

Tomato (*Solanum lycopersicum* L.) is one of the most widely cultivated and consumed crop plants in the world and it is well known for its wide variety of health-promoting components, among which carotenoids are prominent [1]. For instance, carotenoids like lycopene, are important antioxidants which are also responsible for the characteristic red color of tomatoes. Moreover, tomatoes are rich sources of various nutrients, vitamins (calcium, iron (Fe), sodium (Na), potassium (K), magnesium (Mg), vitamin A, and vitamin C) and polyphenols such as anthocyanins and phenol acids [2]. These bioactive substances, when they are implemented into the human diet, may decrease the likelihood of developing cancer or other diseases. Moreover, it is recommended to consume tomatoes and their carotenoid components to protect against cardiovascular diseases and to reduce the risk of developing other conditions including colon, stomach, and rectal cancer [3,4]. In addition to the widespread cultivation of tomato plants and their appeal, the growing global population has compelled the agricultural industry to enhance crop yield to meet the demands of billions of consumers [5]. The task of producing sufficient food for the growing global population has emerged as a significant issue. Furthermore, the agriculture sector encounters numerous challenges, including the need to come up with a method that is both environmentally sustainable and highly effective for cultivating crop plants. The effectiveness of traditional bulk fertilizers is reducing in and they are causing harmful impacts on the environment. The data show that the three most important macronutrients for plant growth, nitrogen (N), phosphorus (P), and potassium (K), are absorbed at rates of 30–35%, 18–20%, and 35–40%, respectively. This means that a large portion of the fertilizers applied in the fields are lost and do not reach their intended locations due to factors such as photolysis, hydrolysis, leaching, microbial immobilization, and degradation [6,7]. The controlled and gradual release of nutrients was identified as one of the main benefits that might be obtained by utilizing nanoparticles as nanofertilizers. In the last decade, more attention has been paid to the use of nanoparticles during the process of plant growth. As proven in many papers, macro- and microelements are crucial for plant growth and development and the usage of nanoforms of those elements may improve their delivery and uptake by plants. Nanofertilizers offer a potential option for enhancing the effectiveness and safety of fertilization. For instance, zinc oxide nanoparticles (ZnO-NPs) are nanomaterials that show promise as a potential nanofertilizer. In general, zinc is an important microelement, essential for plants, playing a crucial role in the regulation of auxins, protein metabolism, glucose production, carbohydrate assimilation and plant defense against pathogens and environmental stressors [6,7]. Due to their physical and chemical properties, they show the potential in agricultural production to increase Zn deficiency, which results in increasing crop yields [8]. Application of nanoparticles as fertilizers during crop production is a promising alternative for sustainable agriculture. The main advantage of their use is their properties, like large surface area, high adsorption capacity, extra infiltration capacity and their appropriate and control kinetics to deliver nutrients at targeted sites causing minimum loss [9]. The nanofertilizers release the nutrients in a controlled manner to response to the reaction to different signals, such as heat, moisture or abiotic stress factors. They may regulate the release of nutrients and deliver the correct quantity required by the crops in suitable proportion and promote productivity while ensuring environmental safety [10]. The inherent application of nanotechnology in modern agriculture is responsible for the improvement in agricultural productivity and better yield crop with a reduction in large amounts of agrochemicals, and at the same time a limitation of both costs of fertilizers and pollution ecosystems [9].

Zn and ZnO are commonly applied in the form of nanoparticles during plant cultivation. A common micronutrient deficiency in soil is zinc deficiency and it was pointed out that it is the fourth most important yield-limiting nutrient after nitrogen, phosphorus and potassium [10]. ZnO nanoparticles have shown enhanced antimicrobial activity against bacterial and fungal species and they promote seed germination, seed vigor, early flowering, chlorophyl content, pod yield and enhanced shoot and root growth [11]. However, extensive use of Zn nanoparticles might lead to them entering the environment, especially affecting the atmospheric and soil ecosystems. As a consequence, this may have a considerable effect on many organisms, including humans [10].

Various research has demonstrated the advantageous impact of applying Zn on the growth of tomatoes. For example, it has been confirmed that the use of Zn is responsible for enhancing many factors such as seed germination, the quantity of fruits per plant, fruit weight and yield [7]. While the presence of Zn particles might affect the growth of tomato plants, the absence of this micronutrient can also have adverse effects on the plant. This phenomenon was well demonstrated in the study conducted by Sardar et al. (2021) [12]. The tomato variety was cultivated on calcareous soil and subjected to a treatment of 30 ppm zinc oxide (ZnO). Due to zinc deficiency in calcareous soils, tomato crop yields were reduced when cultivated in such conditions. The plants treated with zinc minerals had the highest levels of TSS, chlorophyll content, vitamin C, flavonoids, carotenoids, and phenolics concentration, as compared to the control group. Moreover, in the study of Nawaz et al. (2012) [13], it was observed that the maximum number of tomato fruits per plant and total yield of tomato was increased by 100% with the usage of 10 ppm Zn (foliar application) associated supplementation of nitrogen (N) and phosphorus (P), compared to the control. Some investigations were conducted to analyze the impact of Zn NPs or nano-ZnO NPs on tomato plants. Faizan et al. (2019) [14] conducted a study where they found that applying ZnO NPs directly to the leaves at a concentration of 50 ppm resulted in an increase in many growth parameters. The results showed a significant rise in shoot and root length, chlorophyll content, and fruit yield, as well as notable increases in catalase, peroxidase, and superoxide dismutase activity. Additionally, higher levels of lycopene and β-carotene content were observed. In the research conducted by Raliya et al. (2015) [15], tomato plants were treated with ZnO NPs using two different methods: foliar treatment and soil-mediated application. The application of ZnO NPs significantly raised the relative chlorophyll content of tomato plant leaves by both methods of application.

Despite the growing popularity and research focus, there are still certain aspects of nanoparticle usage in agriculture that require more examination. Understanding the mechanism of interactions between nanoparticles and plants is crucial for assessing their beneficial and detrimental impacts on agricultural operations and the environment. However, there is a lack of information regarding the aforementioned trades. Multiple studies have demonstrated that the utilization of NPs can impact various factors associated with the growth and development of plants. The utilization of various nanoparticles (NPs) or their oxide forms occasionally resulted in advantageous outcomes in the tested plants. However, under specific conditions, the plant perceives it as an abiotic stressor. There is also a lack of information concerning the influence of nano-ZnO on the allergenic potential of tomato fruits. Moreover, there is only general information that the method of nanoparticle delivery can influence plant growth, but there are no literature reports on how the method of nanoparticle application affects the parameters of tomato fruit. There is still a scarcity of knowledge on this subject, and further investigations are necessary.

Although nanotechnology is used for development of sustainable agriculture by increasing crop production and protection, it should be emphasized that nanofertilizer delivery during plant cultivation can bring threats to the environment. The main concern of using the NPs is their potential accumulation in ecosystems and entering into the food chain. The application of nanofertilizers raises several safety and ethical issues that need to be solved before commercial application. Detailed studies are required to identify the effect of NPs present in food products on human health. As a consequence, the feasibility, risk assessment and hazard identification need to be identified [16]. Application of nano-ZnO as a potential plant growth improvement can influence soil organisms. Although nano-ZnO is considered insoluble, the presence of citric and oxalic acids in soil enhances their solubility which results in increasing mobility and bioavailability in soil solution [17]. In accordance with Rajput et al. (2020) [18], nano-ZnO shows bacteriostatic effect but there are scientific findings that enzymatic activities of bacterial communities in saline-alkali and black soil can be affected by nano-ZnO [8]. Growing usage of nano-ZnO increases the risk of NPs transferring to the food chain mainly by gastrointestinal tract. Impact of NPs on human health still undergoes testing. Based on recent knowledge, the cytotoxicity of ZnO NPs was induced by oxidative stress and inflammatory response, which depend on size and concentration of NPs [19].

In our previous study, an attempt was made to investigate how the application of nanoparticles (nano-ZnO) combined with conventional fertilizer influences tomato plant growth and development, including the antioxidant potential of cultivated plants [4]. The previous study focused on the green parts of tomatoes. The novelty of the present paper is the analysis of the effect of nano-ZnO on the fruits produced by *Solanum lycopersicum* L. In this study, the influence of nano-ZnO concentration as well as the method of NP application on tomato fruits parameters were determined. The research hypothesis included the assumption that ZnO nanoparticles are a factor causing abiotic stress in tomato fruits and significantly affecting their antioxidant activity and allergen production. Therefore, the analysis of tomato fruit fresh weight, MDA amount, the total antioxidant activity, concentration of lycopene and β-carotene, total sugars content and allergenic potential of tomato fruits harvested after cultivation in the nano-ZnO supplemented soil medium of obtained fruits were determined.

## 2. Results

All factors examined in this study were evaluated in fruits harvested from grown plants. All fruits were fully matured. The plants were treated with ZnO NPs either through soil application (SA) or foliar spraying (FS). Furthermore, the growing plants were fertilized with Biohumus SuperForte, excluding the blank sample. A study was conducted to examine how the use of mixed NPs and standard fertilizer affects the selected parameters in tomato fruits. The research was conducted on the variants presented in the Section 4.

### 2.1. Fruits Fresh Weight

All harvested fruits were divided into groups corresponding to the treatment applied to the plants they were derived from. For instance, fruits from each plant that was foliar sprayed with NPs at dose 50 mg/L were put together and then weighed (Table 1). The highest weight was presented by control fruits (Table 1). For plants under nano-ZnO treatment, independently of the used dosage or the method of NP application, the obtained results were significantly lower than both controls (blank and control). The lowest mass was achieved by fruits derived from plants treated with 150 mg/L NPa via soil application. The value of the fresh weight of those tomatoes was lower than blank and control results by a significant 52% and 68%, respectively. On the contrary, fruits collected from plants under SA 50 treatment obtained the highest mass among the fruits from plants supplied with nano-ZnO. The biomass of SA 50 fruits was increased by approximately 15–23% when compared to other plants treated with NPs, except SA 150 while at this juncture the difference was higher (42%). The mass values obtained by fruits collected from plants under foliar spraying treatment were at a similar level (49.2–54.3 g). The mass presented by fruits harvested from SA 250 plants was close to the results reached by FS plants.

### 2.2. The Content of Lycopene and β-Carotene

**Figure 1:**
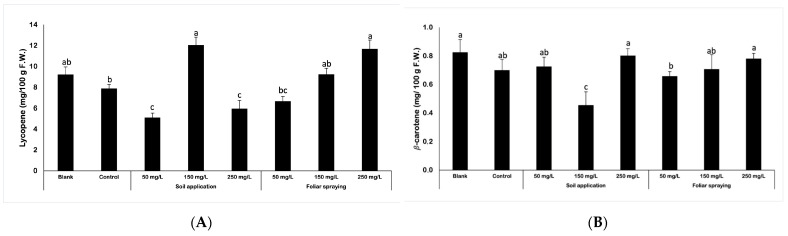
Lycopene [mg/100 g F.W.] (**A**) and β-carotene [mg/100 g F.W.] (**B**) content in tomato fruits of Maskotka cultivar. Vertical bars are used to display all data, which are the means of three replicates (±SD). Different letters (a, b, c, ab, bc, etc.) indicate the significant differences for a *p*-value ≤ 0.05.

The application of nano-ZnO did affect the lycopene content in tomato fruits (Figure 1A). The obtained data indicated that either the used dose of NPs or the method of its application influenced the results. Both controls (blank and control) presented similar levels of lycopene content, though blank fruits contained notably higher lycopene concentration (15%). Higher concentrations of utilized NPs suspension led to an increase in lycopene levels in fruit samples. Using FS dosages of 50, 150, and 250 mg/L resulted in lycopene concentrations between 6.7 and 11.7 mg/100 mL. While the lycopene level reached by FS 50 fruits was lower than control (by 13%), the values reached by FS 150 and FS 250 samples increased by 17% and 48%, respectively. Results presented by plants treated with NPs by soil application demonstrate slightly different trends. The highest score was obtained by plants after 150 mg/L nano-ZnO application. The content of lycopene for those fruits was increased considerably by 53%. Two other dosages of NPs, SA 50 and SA 250 caused contrasting results and exposed decline of lycopene content by 35% and 24%, respectively, compared to the control.

The measurement of β-carotene levels in harvested tomato fruits yielded slightly different results than lycopene analysis, particularly for plants treated with NP by soil application (Figure 1B). The fruits from the SA 150 treatment revealed the lowest β-carotene content. The acquired result was 35% lower than that of control, whereas the SA 50 and SA 250 fruits had β-carotene levels comparable to control. The amount of β-carotene identified in plants whose fertilization was supplemented with nano-ZnO via soil application was correlated with the quantity of NPs applied. However, for plants treated via foliar spraying, the concentration of the utilized suspension did not significantly alter the outcome. FS fruits contain between 0.66 and 0.78 mg/100 mL of β-carotene, which is similar to control. Still, the highest concentration of β-carotene was found in blank fruits, although the difference between blank and other samples (excluding SA 150) is not higher than 20%.

### 2.3. Determination of Antioxidant Activity

**Figure 2:**
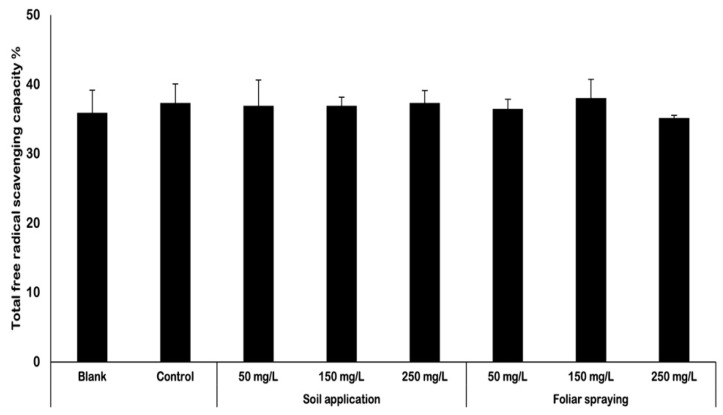
Total free radical scavenging capacity [%] in fruits harvested from Maskotka tomato plants. For statistical analysis, the parametric ANOVA test was conducted. The analyses indicated that there are no statistically significant differences between obtained results.

The total antioxidant activity was assessed using the DPPH method, similarly as measured in plants’ leaves (Figure 2). The results obtained from free radical scavenging capacity (FRSC) conducted in fruits did not vary significantly between each other. The FRSC analysis indicated that results oscillated between 36% and 38%. FRSC values achieved by plants under NP treatment do not vary from both controls (blank and control samples).

### 2.4. Determination of Malonodialdehyde Content (MDA)

**Figure 3:**
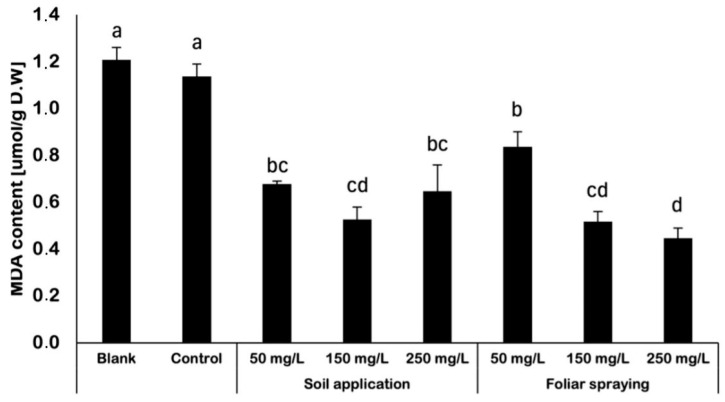
MDA content in fruits harvested from Maskotka tomato plants [µmol per g D.W.]. For statistical analysis, the parametric ANOVA test was conducted. Vertical bars are used to display all data, which are the means of three replicates (±SD). Different letters (a, b, c, ab, bc, etc.) indicate the significant differences for a *p*-value ≤ 0.05.

The analysis of malonodialdehyde (MDA) content displayed significant differences between harvested fruits (Figure 3). Both examined variables, such as the method of NP application as well as the concentration of utilized nano-ZnO suspension affected the obtained results. The highest content of MDA was detected in control and blank samples, range between 1.1 and 1.2 μmol/g D.W. In contrast, the amount of MDA decreased in fruits collected from plants treated with NPs. Among the plants treated with NPs, the highest MDA content was detected in FS 50 samples, and it was still 23% lower when compared to control. With rising NPs suspension concentration, MDA content was decreasing in plants foliar sprayed with nano-ZnO. For plants supplied with NPs via soil application the obtained results were vary between 0.53 and 0.68 μmol/g D.W. For all plants supplied with NPs via soil application, the amount of MDA was significantly lower than for control samples by approximately 38%, 41% and 52% for SA 50, SA 250 and SA 150, respectively.

### 2.5. Determination of Allergen Content

**Figure 4:**
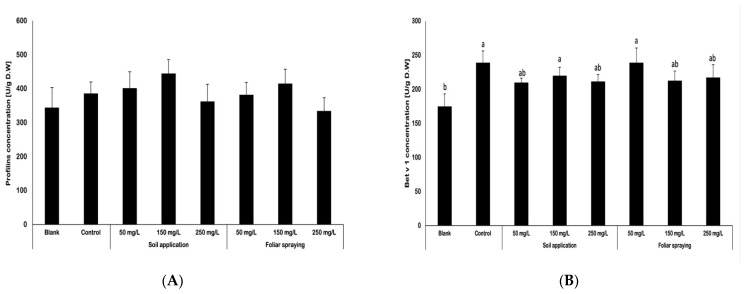
The analysis of profilins (**A**) and Bet v 1 (**B**) content in tomato fruits of Maskotka cultivar. Vertical bars are used to display all data, which are the means of three replicates (±SD). Different (a, b, c, ab, etc.) letters indicate the significant differences for a *p*-value ≤ 0.05.

Tomatoes have been considered as vegetables which may induce an allergic response. Using enzyme-linked immunosorbent assay (ELISA), the concentration of two selected allergens was determined. Profilin, the Sola l 1 allergen in tomatoes, was the first allergen whose presence in raw tomato material was studied (Figure 4A). The obtained conclusions indicate that the implemented features, such as the use of nano-ZnO at varying concentration, applied by dffering techniques, and combined with the use of standard fertilizer, did not significantly affect the profilin content. Nonetheless, slight variations were perceived between the obtained results. The majority of fruits obtained from plants treated with NPs had a notably higher level of profilin in their tissues than each control sample (blank and control). Nevertheless, the lowest levels of profilin were discovered in FS 250 fruit samples. Compared to blank sample and control fruits, the quantity of profilins in FS 250 decreased by less than 5% and 13%, respectively. In the case of the aforementioned results, it is important to indicate that the standard deviation of these data does not allow considering these differences to be statistically significant.

Bet v 1 was the next allergen whose presence was evaluated in the fruit samples (Figure 4B). This allergen is analogous to the Sola l 4 tomato allergen. In the case of this allergen, the obtained results varied notably. Control included the lowest concentration of Bet v 1, whereas control contained a 37% rise in this allergen’s concentration. The results obtained by SA 150 and FS 50 fruits were comparable to those of the control group. But, nonetheless, other applied treatments caused Bet v 1 to be less prevalent in fruits, compared to control. The concentration of Bet v 1 in samples treated by SA with NPs at 50 mg/L and 250 mg/L was 12.2% lower in both, than the amount found in control samples. In the case of foliar spraying, the application of nano-ZnO at concentrations of 150 and 250 mg/L reduced Bet v 1 content. The aforementioned samples contained approximately 10% less in Bet v 1 than in control. Even if the mentioned differences between results did not exceed 15%, it still may suggest that the application of nano-ZnO at specific doses may mitigate the effect of fertilizer on the increase in Bet v 1 content in treated plants.

### 2.6. Determination of Total Sugars Content

**Figure 5:**
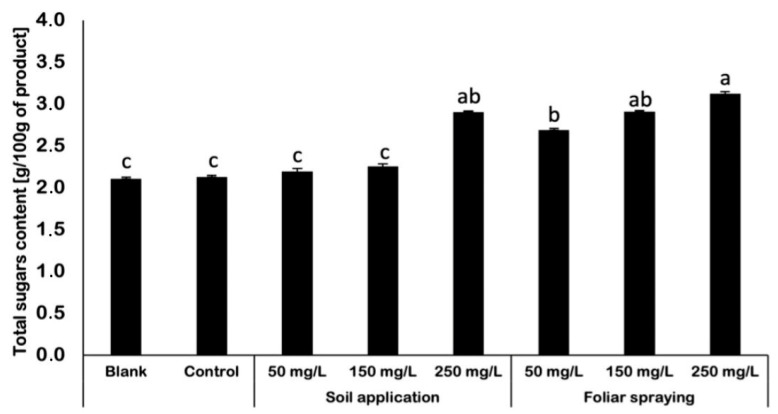
Total sugars content [g/100 g of product] in tomato fruits of Maskotka cultivar. Vertical bars are used to display all data, which are the means of three replicates (±SD). Different letters (a, b, c, ab, bc, etc.) indicate the significant differences for a *p*-value ≤ 0.05.

The determination of total sugars content showed that nano-ZnO affected the sugar content in examined fruits. The highest increase in total sugars content was noticed for foliar-sprayed tomatoes (Figure 5), while for the plants treated with NPs via soil application (except SA 250) the total sugars content was similar to control and blank. For fruits obtained from control plants, the sugar content was equal to 2.1 g/100 g of product. Similar results were obtained by SA 50 and SA 150 plants. A significant increase in total sugars content was noted for plants treated with nano-ZnO via soil application at the highest concentration of NPs (250 mg/L). When compared to control, total sugars content in fruits obtained from SA 250 plants was 38% higher. Moreover, for all plants treated with NPs by foliar spraying, a significant increase in total sugars content was measured/noticed. FS fruits contained from 2.7 to 3.1 g of sugars. The highest result was obtained by FS 250 plants, where fruits contained approximately 48% more sugars than control fruits.

## 3. Discussion

### 3.1. Fruits Fresh Weight

The main aim of using fertilizers and other additives during plant cultivation is obtaining the highest possible yield. Apart from other parameters such as biometric features or antioxidant potential, the fruits yield is the most important aspect of fertilization. In this study, the fruits yield was expressed as the total fresh weight of harvested tomatoes which were divided into groups corresponding to the treatment applied to the plants they were derived from.

Among all harvested fruits, control samples exhibited the highest weight (Table 1). Regardless of the dosage or the method of NP application, the results obtained for plants treated with nano-ZnO were significantly lower than those of the control group (blank and control). Fruits from plants exposed to 150 mg/L NPs via soil treatment had the lowest mass. Interestingly, the fruits harvested from plants subjected to SA 50 treatment exhibited the greatest mass compared to those obtained from plants under nano-ZnO supplementation. The fresh weight values of fruits collected from plants subjected to foliar spraying treatment were found to be comparable. Still, all plants under NP treatment provided lower yield (by weight) than control and blank plants. Furthermore, control plants obtained a higher weight of harvested fruits than blank plants, what confirmed the beneficial aspect of fertilizer usage. Though, it can be assumed that the use of nano-ZnO reduced the nutritional role of fertilizer. The beneficial impact of NPs on tomato yield was presented in the research of Faizan et al. (2019) [14]. According to the authors, the plants that were treated with ZnO NPs exhibited a significant increase in the number of fruits and yield during the harvest period. The foliar application of ZnO NPs at a concentration of 50 ppm resulted in a significant increase in both the number of fruits per plant (21.1%) and fruit yield (19.4%) compared to the control group. However, applying a higher concentration of nano-ZnO suspensions (200 mg/L) to the leaves resulted in a decrease in fruit yield compared to the control. The disparity between the findings of the authors of this study and those provided by Faizan et al. (2019) [14] may be explained by variations in the delivered dosages of nano-ZnO. Furthermore, in our study, the type of NP application had significant impact on the collected data. Although the treatment of NPs resulted in a decrease in fruit output, the rate of reduction varied depending on whether the plants were treated through soil application or foliar spraying. Additionally, in the research of Ahmed et al. (2023) [20] a considerable increase in fruits weight was observed in tomato plants treated with nano-ZnO at 100 ppm dosage (94% higher than control) and followed by plants treated with 125 ppm of ZnO NPs [20]. The findings of both mentioned studies and the results obtained in this research confirmed the undeniable influence of Zn on fruit production. The differences that occur between the abovementioned studies and our research may be the result of using different cultivars in research and different doses of nano-ZnO. These observations are understandable solely due to the fact that Zn is essential for plant nutrition and is thought to be an essential part of several enzyme systems [21]. It has been associated with controlling growth, protein synthesis, energy generation, enzyme activation, gene expression, phytohormone action, photosynthesis, carbohydrate metabolism, fruit production and disease defense [22]. The fact that in the presented research, the total weight of harvested tomatoes was considerably lower than in control plants may be caused by the specific doses of Zn in which plants were supplemented. For foliar-sprayed plants, the direct exposure of plants to NPs during flowering stage could affect the fruits yield. For plants treated with NPs via soil application, the highest yield was achieved by plants exposed to the lowest concentration of NPs, which in this case seems to be the most favorable. The type of ZnO NPs delivery method can influence the plant growth parameters. The soil application delivery method is a method of nutrient supplementation using fertilizers that depends on soil properties. It is demonstrated that the adsorption of mineral nutrients is heavily affected by negatively charged soil particles [9]. According to Mathur et al. (2022) [9] foliar application of nanofertilizer in agricultural crops brings faster nutrient uptake and improves fertilizer movement through the plant in both apoplastic and symplastic pathway, which increases its penetration to plant cell. Therefore, foliar spraying has a positive effect on growth, yield, and physiological parameters of plants [9].

### 3.2. Determination of Antioxidant Activity

The total antioxidant activity in tomato fruits was measured with the use of DPPH method and expressed as free radical scavenging capacity (FRSC). The results obtained from free radical scavenging capacity (FRSC) analysis conducted in fruits did not vary significantly between each other (Figure 2). Based on the results it can be stated that regardless of the method of nano-ZnO application and their concentration, there was no significant effect of nano-ZnO on free radical scavenging capacity in tomato fruits. Nevertheless, it is important to consider that examined fruits derived from plants under soil application of NPs, were not exposed directly to nano-ZnO. Interestingly, in foliar spraying, fruits were directly exposed to the nanoparticles, although this exposure was limited. On the contrary, in the study of Ahmed et al. (2023) [20], the scavenging activity measured by the DPPH assay in plants treated with nano-ZnO at doses of 75, 100, and 125 ppm (mg/L) and nano-Zn at a dose of 1500 ppm (mg/L) increased significantly. The disparities seen in our study compared to the abovementioned research conducted by Ahmed et al. (2023) [20] can be attributed to various factors. Firstly, there are variations in agronomic practices between the two research (fertilization and environmental conditions). A further difference is the choice of tomato cultivar. In each investigation, the authors studied three different tomato cultivars. Moreover, there were notable differences in the foliar application of NPs between the two investigations. In the research of Ahmed et al. (2023) [20], only three separate foliar applications of nano-ZnO were undertaken at different stages of plant growth. In our research, plants were sprayed consistently every 2 weeks. The mentioned factor could significantly affect the results obtained in both studies. Though, in the investigation of the impact of ZnO NPs on the candy leaf (*Stevia rebaudiana* L.) conducted by Ahmad et al. (2020) [23] they reported an increase in % DPPH inhibition by 76% due to the treatment with 2 mg/L of nano-ZnO. However, the DPPH assay is used to predict antioxidant activities by mechanism in which antioxidants act to inhibit lipid oxidation, so scavenging of DPPH radical and therefore determinate free radical scavenging capacity. The method is widely used due to the relatively short time required for the analysis. Although often used, this tool is inaccurate and insufficient for showing the precise antioxidant activity. Further investigations of plants’ response to nano-ZnO were required to show the real picture of changes that occur in their tissues. Due to the discrepancy between the results of total antioxidant activity and the concentrations of specific antioxidants, i.e., lycopene and β-carotene, an MDA test was performed to completely confirm or exclude the hypothesis that nano-ZnO may be a factor of abiotic stress and cause an increase in antioxidant activity. MDA test is a widely used marker of oxidative lipid injury caused by environmental stress. In this test membrane lipid peroxidation in plants is detected by measuring malondialdehyde.

### 3.3. Determination of MDA Content

Malondialdehyde (MDA) is a byproduct produced by lipids in cell membranes in response to reactive oxygen species, and it serves as a marker for oxidative stress. MDA content is a frequently used indicator of lipid peroxidation in plant tissue, which rises in reaction to oxidative stress. This value is regarded as a biomarker for oxidative stress. The analysis of fruits obtained from NP-treated plants showed an opposite effect. The usage of nano-ZnO during cultivation led to the reduction in MDA content in examined fruits. The highest MDA content was detected in control fruits. In the research of El-Zohri, Al-Wadaani and Bafeel (2021) [24], the implementation of nano-ZnO helped to inhibit the damaging results of drought on tomato plants. Application of ZnO-NPs through foliar spray at the specific doses (25 and 50 mg/L) resulted in a decrease in the formation of MDA and H_2_O_2_ in tomato leaves when subjected to drought conditions at 75%, 50%, and 25% of field capacity, as compared to the control group that did not receive any treatment. The usage of ZnO-NPs (at concentration of 100 mg/L) did not have a significant impact on the MDA concentration at any of the drought levels, compared to their respective control samples [24]. Moreover, in a study conducted by Sun et al. (2020) [25], it was shown that treating maize leaves with ZnO-NPs resulted in reduced levels of H_2_O_2_ and MDA. This treatment helped to prevent lipid peroxidation caused by drought and maintained the stability of the plasma membrane. The study conducted by Amooaghaie, Norouzi, and Saeri (2016) [26] investigated the MDA content in tomato plants treated with nano-ZnO. This study found that the tomato plants noticed a considerable rise in MDA content when exposed to a nano-ZnO suspension at a concentration of 100–200 mg/L. Unfortunately, the authors provided only a restricted amount of data for this research, as they only published the results of plants that were treated with suspensions at concentrations of 100 and 200 mg/L. The absence of results achieved for plants subjected to alternative dosages of NPs impeded the comprehensiveness of the study. Notably, in all abovementioned studies, the MDA content was evaluated in green parts of plants. To the best of the authors’ knowledge, there are limited data concerning the analysis of tomato fruits under NP treatment. In general, data currently available indicate that the application of nano-ZnO has varying effects on plants depending on the cultivation conditions, concentration of applied NPs suspensions and the choice of examined plants’ tissues.

### 3.4. The Content of Lycopene and β-Carotene

Lycopene is a pigment primarily responsible for the dark red color of ripe tomato fruits. It is an interesting and desired compound by humans because of its biological and physicochemical properties, while it is also a natural antioxidant. In addition to this nutrient, tomato fruits also contain β-carotene. Both people and animals are devoid of carotenoid biosynthesis, but if they absorb β-carotene from plants food, they are capable of transforming it further into retinol (vitamin A). The analysis of carotenoid content in tomato fruits was conducted by the evaluation of lycopene and β-carotene content. Interestingly, the obtained results were strongly correlated with the results of fruits yield. The highest concentration of lycopene was detected in fruits from plants exposed to NPs at dose 150 mg/L applied through soil. In comparison to the control, the lycopene concentration in SA 150 fruits was 53% higher. On the contrary, fruits collected from plants treated with 50 and 250 mg/L NPs demonstrated significantly decreased lycopene content when compared to the control. For plants foliar sprayed with NPs, increasing concentrations of nano-ZnO suspension were related with raising concentrations of lycopene in fruits. Nonetheless, the usage of 150 and 250 mg/L suspension caused a lycopene content considerably higher than in control plants. In the research of Faizan et al. (2019) [14], the beneficial impact of NPs on the lycopene content was demonstrated. Plants treated with ZnO NPs yielded fruits with elevated levels of lycopene in comparison to the control group. The plants exhibiting the highest concentration of lycopene were exposed to 50 ppm of nano-ZnO, while the observed increment was equal to 22.6% in comparison to the control group. In the research of Raliya et al. (2015) [15], the gathered fruits obtained from the treated plants showed an increase in lycopene concentration in comparison to the control plants, except for the plants that were treated with a foliar application of 1000 mg/kg ZnO NPs. The induction of lycopene biosynthesis was higher via foliar application as compared to soil application. Moreover, when compared to the control, the lycopene concentration exhibited a significant increase by 113% in fruits derived from plants treated with foliar application of 100 mg/kg ZnO NPs. Another study in which the nano-ZnO application increased lycopene content in tomato fruits was presented by Ahmed et al. (2023) [20]. The authors proved that the foliar application of nano-ZnO at doses 75, 100 and 125 ppm led to an increase in lycopene content, while an increased dose of Zn foliar application caused a decline in the lycopene content. The β-carotene content in examined tomato fruits depended on both the utilized dose of NPs and the method of its application. In plants under foliar spraying treatment, β-carotene content was increasing with the increasing concentration of NPs suspension. For plants which were supplemented with NPs through soil, the lowest concentration of β-carotene was detected in fruits from plants treated with 150 mg/L dosage of nano-ZnO. On the other hand, in fruits derived from plants supplied with NPs at doses 50 and 250 mg/L, β-carotene content was considerably increased. Interestingly, those results are in the opposite trend when compared to lycopene content. The occurrence of this issue may be attributed to the activity of lycopene β-cyclase (LCYB) in plant cells, which converts lycopene into β-carotene [27]. The decreased concentration of lycopene and increased concentration of β-carotene may be attributed to the aforementioned enzyme. The administration of specific doses of nanoparticles (NPs) may have indirectly led to an increase in the activity of LCYB. In the research of Faizan et al. (2019) [14] the beneficial effect of foliar spraying with nano-ZnO was reported. One of the parameters that was affected by nano-ZnO was the β-carotene concentration. The authors have reported that the utilization of NPs at 50 ppm dose caused a significant increase in β-carotene concentration (by 25%). Based on those findings and the results obtained in this study, it can be concluded that nano-ZnO is the compound that affects the concentration of β-carotene in tomato fruits. In contrast to those observations, the application of Se NPs and Cu NPs, combined or separately, did not affect noticeably the β-carotene concentration in tomato fruits as reported by Hernández-Hernández et al. (2019) [28]. Moreover, the co-exposure of Se NPs at a concentration of 10 mg/L and Cu NPs at a concentration of 10 mg/L, along with all other doses of Cu NPs, resulted in a reduction in the lycopene content in tomato fruits. In addition, in the research conducted by González-Moscoso et al. (2019) [29], they analyzed the influence of Si NPs on antioxidants in tomato plants stressed by additives of arsenic. The β-carotene content was reduced by 24% in plants exposed to 250 mg/L of SiO_2_ NPs. Moreover, β-carotene concentration was decreased by 40% due to the interaction between 3.2 mg/L arsenic and 250 mg/L SiO_2_ NPs. The use of 1000 mg/L of SiO_2_ NPs in the absence of arsenic resulted in a 13% increase in the β-carotene concentration.

### 3.5. Determination of Allergen Content

Tomatoes have been considered as vegetables which may induce an allergic response. It is important to evaluate if the cultivation methods connected with use of fertilizers, specific growing condition or exposure to nanoparticle may affect the allergenic potential of grown tomatoes. The analysis of selected allergens (profilins and Bet v 1) content in harvested tomatoes was carried out with the use of enzyme-linked immunosorbent assay (ELISA). The results obtained suggested that the variables that were implemented, including the utilization of nano-ZnO at different concentrations, applied through different methods, and in combination with conventional fertilizer, did not have a significant impact on the profilin content. However, there were slight variations observed among the acquired results. It was observed that fruits derived from plants subjected to NP treatment exhibited an elevated concentration of profilin in their tissues compared to both the control and blank samples. The results obtained for Bet v 1 exhibited variation, with the lowest concentration of this allergen detected in control. The blank samples exhibited a lower concentration of Bet v 1 in comparison to the control tomatoes. This observation may indicate that the application of fertilizer contributed to the elevation of Bet v 1 concentration in tomatoes. The outcomes acquired from fruits treated with NP treatment at a concentration of 150 mg/L through soil and 50 mg/L via foliar spraying exhibited similarity to the control group. However, other doses resulted in a decrease in the abundance of Bet v 1 in fruits as compared to the control group. Even though the differences between the obtained results of Bet v 1 content in examined fruits was minor and it is not possible to state that the use of NPs had the direct influence on those findings.

To the best of the author’s knowledge, this is the first research in which the impact of nanoparticles on the allergenic potential of cultivated plants was investigated. Only a few studies evaluate the influence of cultivation conditions on the allergenic potential of tomato fruits. Kurze et al. (2018) [30] investigated how the features such as tomato color, size and shape or the cultivation method and processing techniques were correlated with the quantity of Sola l 4. The study found that among 23 different varieties of tomatoes, content of Sola l 4 varied significantly between 0.24 and 1.71 μg Sola l 4/g FW. The process of drying tomatoes through either oven or sun exposure has led to a notable reduction in Sola l 4 in processed fruits. The study confirmed the thermal instability of the recombinant Sola l 4 protein, with particular emphasis on the findings related to the native protein present in dried tomato samples. In general, the amount of Sola l 4 content is dependent on the cultivar, and there was no observed correlation between color and the quantity of Sola l 4. The thermal instability during the drying process of tomatoes resulted in a significant reduction in Sola l 4 level. The impact of growing conditions is relatively insignificant, while seasonal effects exhibit a more notable influence [30]. Those findings confirm the concentration of Sola l 4 may fluctuate due to different factors. It is worthy to note that in this research that the lowest amount of Bet v 1 (Sola l 4 analogue) was detected in blank samples, which indicate that the utilization of fertilizer affected this parameter. Though, the exposure to NPs led to a decrease in Bet v 1 concentration (except foliar spraying with NPs at dose 50 mg/L). Słowianek et al. (2016) [31] carried out a more in-depth study with a focus on the cultivation method. The abovementioned authors conducted a 3-year long investigation on the presence of a few selected allergens (Profilin, LTP, and homolog of Sola l 4) in tomatoes that were grown separately in organic and conventional systems. Additionally, both growing methods were used in this study with five distinct cultivars. According to the data, there is a statistically significant link between allergen levels in fruits and cultivar for each allergen that was studied. However, the only allergen, for which there was a link between the allergen levels and the cultivation method, was profilin. Tomatoes from organic farming practice in this instance had a greater allergenic potential [31].

### 3.6. Determination of Total Sugars Content

Sugars in fruit contribute to its sweetness, which is the primary factor influencing fruit quality. The correlation between the amount of sugar in fruits and the quantity of fruit produced has been assessed in many horticulture investigations [32]. The obtained data showed that implementation of additive zinc in form the of nanoparticles influenced the content of sugar in some tomato fruits. The rise of sugars content was noticed mainly in tomatoes which were foliar sprayed with nano-ZnO. The increased amount of total sugars may be attributed to the stress induced by direct exposure of fruits to nanoparticles (NPs). In a study conducted by Cantu et al. (2022) [33], the use of ZnO NPs resulted in a 100% rise in sugar concentration compared to the control group. The study conducted by Boulouri and van den Ende (2012) [34] revealed that the sugar content plays a significant role in stress-related responses.

Overall, zinc serves as a crucial component or regulatory co-factor in various enzymes and proteins within plants. It is particularly involved in important biochemical pathways related to carbohydrate metabolism (including photosynthesis and sugar-to-starch conversion), protein metabolism, auxin metabolism (a growth regulator), and pollen formation [35]. Zinc is a crucial element involved in carbohydrate metabolism by activating specific enzymes such as carbonic anhydrase, fructose-1, 6-bisphosphate, and aldolase [36]. This phenomenon may explain the increase in total sugars content in fruits which were directly exposed to nano-ZnO. It was reported that the usage of nano-ZnO increased significantly the concentration of soluble sugars in *Rosmarinus* [37]. The largest amount of soluble sugar was observed in plants treated with nano-ZnO at concentration of 7 g per thousand. The results of this study indicate that raising the levels of soluble sugars in plant shoots affects the response to zinc treatment and nano zinc oxide treatment. The authors of abovementioned findings Mohsenzadeh and Moosavian (2017) [37], have established that obtained results may lead to conclusion that the potential for an increase in the concentration of soluble sugars in the shoot lead to an increase in the transmission of soluble sugar to the root. This transmission serves to uphold the root’s essential activities and cellular osmotic adjustment. The authors provided evidence to support their conclusions by showing that under stressful conditions, the accumulation of soluble sugars serves to not only maintain osmotic potential, but also ensure that glucose reserves for basic cellular metabolism are maintained at an adequate level [38]. Although, regarding the results obtained in this study, the decrease in MDA content may indicate that fruits obtained from plants under NP treatment did not perceive the exposure on NPs as a stressor. This may lead to the conclusion that the rise in sugars content may be connected with the role of zinc in carbohydrates metabolism. In another study, provided by Davarpanah et al. (2016) [38] the usage of nano-Zn and nano-B (nano-boron) was also linked with significant increase in total sugars in pomegranate fruit. Regarding this study the trees treated with the maximum concentration of combined Zn and B had the highest amount of total sugars. On the other hand, the control untreated trees had the lowest level of total sugars. The study of Davarpanah et al. (2016) [38] can again indicate that Zn affects total sugars by influencing starch and nucleic acid metabolism, as well as the activities of enzymes involved in these biochemical pathways. On the other hand, the effects of B on total sugars can be attributed to its role in sugar transport and carbohydrate metabolism. The abovementioned studies cannot be fully compared while in each study different plants and different forms and concentrations of nano-Zn were evaluated, but the common outcome for them is the significant increase in sugars in examined samples. Those findings may encourage further research providing more data on that topic, since implementing nano-ZnO or nano-Zn in plant cultivation may possess potential in boosting desired feature of fruits and vegetables without causing strong abiotic stress.

## 4. Materials and Methods

For this research cherry-type tomato cultivar Maskotka (W. Legutko) (*Solanum lycopersicum* L.) was used. Seeds of selected are commercially available.

ZnO NPs suspensions were prepared of NPs < 50 nm (Sigma Aldrich, Saint Louis, MO, USA) with varying concentrations (50, 150, 250 mg/L) in deionized water and dispersed by ultrasonic vibration (100 W, 40 kHz) for 30 min to avoid aggregation (Bandelin Sonorex DT 102 H). The characteristics of ZnO NPs are demonstrated according to the reports of Sigma Aldrich company as follows: particle size ZnO, 50 nm, purity 97.0% and surface area >10.8 m^2^/g [4].

The cultivation was provided in different variants as follows: blank sample (soil medium without fertilizers), control (soil with fertilizer, no NPs added), soil with application of NPs in different concentration and fertilizer (marked as SA 50, SA 150, SA 250) and soil with fertilizer and NPs in different concentrations applied as foliar spraying (marked as FS 50, FS 150 and FS 250). And 50, 150 and 250 indicate nano-ZnO concentrations.

### 4.1. Cultivation

Seeds were sown on prepared soil medium (blank sample), soil medium enriched with conventional fertilizer Biohumus SuperForte (Agrecol J.P., Mesznary, Poland)—control sample and the same soil medium supplemented by conventional fertilizer with ZnO NPs (at size < 50 nm) suspension at three different concentrations (50, 150, and 250 mg/L). The concentration of ZnO NPs used in the study was chosen in accordance with scientific reports to observe the significant effect of minimal ZnO NPs concentration on plant growth parameters as well as the high concentration ZnO NPs impact on tomato fruits growth [8,15]. Each plant received a total of 300 mL of fertilizer throughout 6 months, providing approximately 0.3 mg of nitrogen (N), 0.15 mg of phosphorus (P), and 0.3 mg of potassium (K). Plants treated with nanoparticles were given specific amounts of zinc supplementation, those treated with 50 mg/L of nano-ZnO received approximately 9 mg of zinc, while those treated with 150 mg/L received 28 mg, and those treated with 250 mg/L received 46 mg of zinc. For convenience, the phrases 50, 150, and 250 mg/L solution/suspension appear in the following sections, although they refer to the amounts of Zn given above. All plants were fertilized (50 mL), selected plants (FS) were sprayed with the NPs solution every two weeks, and other plants were treated with the NPs solution via soil approximately once per month (30 mL).

All implemented factors and parameters of cultivation were described in our previous work [4]. During the experiment, two methods of ZnO NP application were used (soil and foliar spraying) to compare the impact of ZnO NPs method utilization on plant growth parametres.

There were three replicates of each treatment. After cultivating Maskotka tomato plants for 6 months, the mature fruits were harvested. Every fruit was picked when it reached full ripeness. The Maskotka fruits were small, red and cherry type. All collected fruits were similar in size and showed no significant variation. Before being subjected to further investigation, the samples were homogenized and stored at −20 °C.

### 4.2. Determination of Antioxidant Activity (DPPH)

The total antioxidant activity in fruits was determined using the DPPH method and quantified as free radical scavenging capacity (FRSC). Antioxidants were extracted from the samples by mixing 2 mL of methanol with 0.05 g of grounded with the use of mortar and pestle fruits tissue [39]. Samples were kept for 48 h at 4 °C and after that period they were placed in an ultrasonic bath for five min at 27 °C. Next, samples were then centrifuged for 10 min at 16,000× *g*. (Centrifuge type Sigma 2-16P, Polygen, Poland). The 300 mM of DPPH solution was prepared through dilution in 80% methanol (*v*/*v*). A volume of 100 μL of plant extract was added to 1 mL of DPPH solution and 3.9 mL of 80% methanol. The absorbance was measured every after 5, 15 and 30 min of incubation against blank at 517 nm (UV/VIS8453 Spectroquant Nova 400 spectrophotometer (Merck KGaA, Darmstadt, Germany)). All analyses were performed in triplicate. The DPPH radical scavenging activity was calculated from the following equation:

RSC [%] = 100 (A0 − A)/A0

where A was an average absorbance of the sample and A0 referred as an average absorbance of a control (DPPH).

### 4.3. The Content of Lycopene and β-Carotene

An amount of 0.1 g of fruit was added to 1 mL of an acetone-hexane solution (4:6) and vortexed. The mixture was then centrifuged at 1500× *g* for 5 min at 4 °C. The absorbance at 663, 645, 505, and 453 nm was determined using an aliquot of the upper phase (UV/VIS8453 Spectroquant Nova 400 spectrophotometer (Merck KGaA, Darmstadt, Germany)). According to the Nagata and Yamashita (1992) [21] formula, the amounts of lycopene and β-carotene were determined and represented as g/100 g F.W.

### 4.4. Determination of Allergen Content (ELISA Assay)

By using an indirect, non-competitive approach called ELISA, the possible allergenicity was assessed. In order to identify Bet v 1 analogues, mouse anti-Bet v1 antibodies ([BV16], Merck, Darmstadt, Germany) were initially utilized. To determine the presence of a protein-like profilin, the mouse anti-profilin antibodies (Anti-Profilin-1 [mAbPRF1a], Agrisera, Sweden) were also used. The secondary antibodies against the mouse immunoglobulin conjugated with alkaline phosphatase (Alkaline Phosphatase-conjugated Affinity Purified Goat anti-Mouse IgG (H + L), Sigma-Aldrich, Poznań, Poland) were utilized. The 3% solution of commercial skim milk was used to block the wells on the plates. Alkaline phosphatase pNPP (Merck, Germany) was used as the substrate, 3 M NaOH (Sigma-Aldrich, Poland) was used as the stop reagent, and PBS with 0.1% Tween 20 was used as the washing agent. A standard curve made with the Bet v 1 allergen or profilin was used to determine the results after the absorbance was measured at 405 nm using a Multiscan RC microplate reader (Labsystems, Vantaa, Finland).

### 4.5. Determination of Total Sugars Content

The SCALES method was utilized to determine the total sugar concentration. An amount of 2 g of fresh tomato tissues was mixed with 25 mL of distilled water. Subsequently, a solution of 30% HCl was added to the tomato solution. The mixture was then subjected to a water bath maintained at a temperature of 68 °C for a period of 5 min. Following incubation in a water bath, the flask was rapidly cooled and then neutralized with a 2 M NaOH solution against a drop of methyl orange. This solution served as a stock solution for measuring the total sugars (Rcog). Next, a mixture of 10 mL of Fehling I and 10 mL of Fehling II solutions was combined with 20 mL of the stock solution to determine the concentration of reducing sugars. The solution was heated to its boiling point and maintained at that temperature for 2 min. In order to dissolve the CuO precipitate, a solution of 10% HCl was introduced into the heated mixture together with 8% NaHCO_3_. The flask with mixture was cooled until it reached room temperature. Right after the cooling procedure was complete, a 0.1 M iodine solution was put into the flask. The flask was then sealed and placed in the dark for 5 min. Following this time, a starch solution was used as an indicator, and excess iodine was measured by titrating it with a standardized solution of sodium thiosulfate. At the conclusion of the titration, when the solution’s color becomes significantly lighter. Based on the amount of iodine, read the amount of invert sugar in the test sample from the table for the SCALES method.

### 4.6. The Content of Malonodialdehyde (MDA)

To determine malondialdehyde (MDA) content in leaves, the 2-thiobarbituric acid (TBA) in accordance with the method of Li et al. (2013) [22] was applied. The first step of the procedure was to ground 1 g of plant tissues with 5 mL 10% trichloroacetic acid (TCA). Subsequently, the mixture was centrifuged at 4000× *g* for 20 min and 2 mL of supernatant was mixed with 2 mL of 0.6% thiobarbituric acid in 10%TCA. Next, the sample was heated at 100 °C for 15 min and then cooled in ice. Lastly, samples were centrifuged at 4000× *g* for 10 min. The absorbance was measured at 450, 532, and 600 nm. Data were calculated as μmol per gram of dry weight (μmol/g D.W.). The MDA content was determined on a dry weight basis as follows:

MDA (μmol/g D.W.) = 6.45(A_532_ − A_600_) − 0.56A_450_

### 4.7. Statistical Analysis

Statistical analysis was provided by ANOVA tools in accordance with description provided by Włodarczyk et al. (2023) [4]. The data were represented as the mean value ± the standard deviation and statistically evaluated using R Studio. The statistical significance of the treatments was assessed using two or three component analysis of variances (ANOVA), conducted on the investigated parameters. An analysis of variance was conducted to assess the significance of differences between the means of different variants in the experiment. The observed disparities were deemed statistically significant when the *p*-value was below 0.05, as determined by either the Tukey or Dunn statistical test. In this context, distinct letters allocated to means indicate a statistical distinction at a *p*-value of 0.05 or less. The selected data were analyzed using non-parametric statistics based on the results obtained from descriptive analysis. The means were compared using the Kruskal–Wallis test, which revealed significant differences.

## 5. Conclusions

In this study, we investigate the effects of various fertilization methods, including the application of nano zinc oxide (nano-ZnO), on the yield and quality of tomato fruits, as well as their allergenic potential. This study reveals that nano-ZnO application in tomato cultivation affected selected parameters. While control samples consistently exhibited the highest fruit yield, plants treated with nano-ZnO displayed lower yields across all dosages and application methods. This study found no significant variation in antioxidant capacity (measured as free radical scavenging capacity) among different treatments, except for variations related to the concentration and the application method of nano-ZnO. Direct exposure of fruits to nano-ZnO through foliar spraying did not significantly affect total antioxidant capacity. Interestingly, even though the total antioxidant activity was not significantly affected by the NPs usage, the MDA content which is directly correlated with the plants’ reaction on stressors was notably decreased. The nano-ZnO utilization caused a significant decrease in MDA concentration in all fruits under NP treatment. Those findings may suggest that the use of nano-ZnO was not considered as abiotic stress for examined fruits. Moreover, lycopene concentration varied based on dosage and application method, with some instances of increased content. β-carotene content exhibited an opposite trend to lycopene. Regarding allergenic potential, nano-ZnO treatments had no significant effects on profilin content but had slight impact on Bet v 1 concentration, although the differences among the results obtained for each treatment were minor, which may be considered as beneficial effect of NPs usage. Nano-ZnO treatment led to an increase in total sugars content in foliar-sprayed tomato fruits, which can be considered a positive effect on fruit quality. These findings highlight the complexity of nano-ZnO’s effects on tomato cultivation, creating the need for further research to optimize its application for more advantageous outcomes. Nano-ZnO application, while potentially beneficial for certain aspects such as lycopene or total sugars content, may not always improve fruit yield, and could have various effects on antioxidant capacity and allergenic potential. The dosage and the method of nano-ZnO application play a crucial role in determining its effects on tomato plants. In this study, three different concentrations of nano-ZnO were tested to find the optimal dose of reagent that influences tomato growth. Due to the possibility of including nano-ZnO to the trophic chain and unknown precise influence on living organisms, the dosage of nano-ZnO should be the lowest that ensures the improvement of crop or consumer plant quality. Moreover, the economic aspect should be also taken into account before determination of the optimal nano-ZnO dose. In the present study, the commercially available nano-ZnO was used. Such a reagent may be economically excluded due to its higher costs when compared with traditional fertilizers. However, the amount of nanofertilizers recommended as fertilizers is lower when compared to traditional fertilizers, which results in a more cost-effective process. To overcome this problem and reduce the total costs of nano-ZnO in large-scale treatment, nano-ZnO can be synthetized from green plants in amounts needed for specific cultivation. Further research is needed to optimize nano-ZnO application for desired outcomes in tomato cultivation.

Future research should include the toxicological studies of nano-ZnO. Although the nano-ZnO treatment showed no negative impact on the quality of tomato fruit, it should be emphasized that usage of NPs in agri-food industries may influence human health, especially in the context of allergenicity. The current state of knowledge is that nano-ZnO shows different degrees of toxic effects on human A549 cells, human gingival cells, zebrafish cells, HepG2 cells and human endothelial cells [40]. The cytotoxicity of nano-ZnO can cause ROS-mediated damage to cellular structures primarily through the production of chemicals and/or damage of cellular structures through physical means, mainly mediated by ROS [40]. Nano-ZnO are taken up by cells, enter the cell, produce ROS, cause intracellular oxidative stress response, and damage DNA, protein, and cell membrane, as well as mitochondrial membrane permeability, leading to apoptosis or necrosis [40]. Therefore, risk assessment should be carefully provided to prove that a low concentration of nano-ZnO is beneficial for plant growth and did not bring negative consequences to human health or any living organisms. Before risk assessment research, it is not possible to include nano-ZnO in crops and/or consumer plant production practices.

The results pointed out in the present study confirm that the analysis should be continued. Future studies are planned to be focused not only on the analysis of the impact of the environmental conditions on tomato growth but also on the dose of nano-ZnO that has a positive effect on tomato fruit quality, and most importantly, the risk assessment of nano-ZnO applications on ecosystems and human health.

## Figures and Tables

**Table 1:** Fresh weight of harvested Maskotka tomato fruits.

Sample	Fresh Weight [g]	The Precentage Representation of Certain Fruits Weight from All Harvested Fruits [%]
Blank	76.2	15
Control	114.0	23
SA 50	63.7	13
SA 150	36.8	7
SA 250	53.8	11
FS 50	49.1	10
FS 150	49.2	10
FS 250	54.3	11

## Data Availability

The original contributions presented in the study are included in the article, further inquiries can be directed to the corresponding author.
